# Emotional enhancement of immediate memory: Positive pictorial stimuli are better recognized than neutral or negative pictorial stimuli

**DOI:** 10.2478/v10053-008-0121-1

**Published:** 2012-08-21

**Authors:** Hanna Chainay, George A. Michael, Mélissa Vert-pré, Lionel Landré, Amandine Plasson

**Affiliations:** Laboratoire d’Etude des Mécanismes Cognitifs, Université Lumière Lyon 2, France

**Keywords:** emotional memory enhancement, explicit/ implicit retrieval, intentional/ incidental encoding

## Abstract

We examined emotional memory enhancement (EEM) for negative and positive pictures while manipulating encoding and retrieval conditions. Two groups of 40 participants took part in this study. Both groups performed immediate implicit (categorization task) and explicit (recognition task) retrieval, but for one group the tasks were preceded by incidental encoding and for the other group by intentional encoding. As indicated by the sensitivity index (*dʹ*), after incidental encoding positive stimuli were easier to recognize than negative and neutral stimuli. Participants’ response criterion was more liberal for negative stimuli than for both positive and neutral ones, independent of encoding condition. In the implicit retrieval task, participants were slower in categorizing positive than negative and neutral stimuli. However, the priming effect was larger for emotional than for neutral stimuli. These results are discussed in the context of the idea that the effect of emotion on immediate memory enhancement may depend on the intentionality to encode and retrieve information.

## Introduction

Memory for emotional information is usually better than memory for neutral information (e.g., LaBar & Cabeza, 2006; Phelps, Spencer, & LaBar, 1997). Several studies that used different types of stimuli (such as words, pictures, or sentences) demonstrated an emotional enhancement of memory (EEM; for a review, see Buchanan & Adolphs, 2002; Hamann, 2001). The EEM effect has been documented in behavioral (e.g., Burke, Heuer, & Reisberg, 1992; Denburg, Buchanan, Tranel, & Adolphs, 2003; Doerksen & Shimamura, 2001; MacKay et al., 2004), neuropsychological (e.g., Adolphs, Cahill, Schul, & Babinsky, 1997; Burton et al., 2004; Hamann, Cahill, McGaugh, & Squire, 1997; LaBar, LeDoux, Spencer, & Phelps, 1995), and neuroimaging (e.g., Döhnel et al., 2008; Hamann, Ely, Grafton, & Kilts, 1999; Richardson, Strange, & Dolan, 2004) studies. In the majority of these studies, the EEM was observed for negative stimuli as compared to neutral stimuli (e.g., Anderson, Yamaguchi, Grabski, & Lacka, 2006; Kensinger, Garoff-Eaton, & Schacter, 2007; Kensinger & Schacter, 2007). In some studies, however, EEM was also reported for positive stimuli (e.g., Boller et al., 2002; Ochsner, 2000; Talmi, Schimmack, Paterson, & Moscovitch, 2007).

The EEM was most frequently shown in tasks involving a long delay between an initial study phase and a later memory test (e.g., Kensinger & Corkin, 2003; Ochsner, 2000; Talmi & Moscovitch, 2004). It has been suggested that EEM is due to a better consolidation of emotional memory traces than that of neutral stimuli, and that this could be related to the modulatory effect of the amygdala on the hippocampus during consolidation (for reviews, see McGaugh, 2004; Phelps, 2004). However, it is not clear whether EEM occurs during encoding, consolidation, or rehearsal. It seems that the emotional nature of a stimulus may affect encoding (Doerksen & Shimamura, 2001; Kensinger & Corkin, 2003), as well as rehearsal (Christianson & Engelberg, 1999) or consolidation (McGaugh, 2000, for a review) of information. From an anatomical perspective, the amygdala seems to be a plausible candidate for such an enhancement since neuroimaging studies have frequently shown that the amygdala is activated during the encoding of emotional stimuli (Dolan, 2002; Maddock, Garrett, & Buoncore, 2003) and that this activation is proportional to the probability of retrieving emotional information (Canli, Zhao, Brewer, Gabriele, & Cahill, 2000). The role of the amygdala in emotional enhancement is also supported by the absence or reduction of such enhancement in participants with amygdalar lesion (Adolphs, Cahill, Schul, & Babinsky, 1997; Adolphs, Tranel, & Denburg, 2000; Buchanan, Denburg, Tranel, & Adolphs, 2001).

EEM was also observed in studies involving immediate retrieval or retrieval after a short time delay, varying from a few seconds to several minutes (e.g., Dolcos & Cabeza, 2002; Dolcos, LaBar, & Cabeza, 2004; MacKay et al., 2004; Talmi, Anderson, Riggs, Caplan, & Moscovitch, 2008; Talmi et al., 2007). It has been pointed out that the modulation hypothesis (amygdala regulation of processing in the hippocampus and striatum during consolidation of memory traces) could not account for the EEM observed in these studies (Talmi et al., 2007). In fact, because of the short delay between encoding and retrieval, it is unlikely that the consolidation would occur, as it takes time for the memory of a stimulus to become “set” (Phelps, 2004). Studies that investigate EEM with different time delays between encoding and retrieval are very helpful for understanding the nature of the EEM and the stage at which this phenomenon occurs. Talmi et al. (Talmi & McGarry, 2012; Talmi et al., 2007, 2008) proposed that the EEM observed on immediate recall or after short delays may be the result of the different involvement of attention. The idea is that the amygdala response to emotional stimuli results in more attention being paid to these stimuli and, thereby, ameliorates their encoding. Better encoding of a stimulus may enhance its’ memory trace and consequently improve subsequent recognition. This suggestion is supported by the observation that emotional stimuli automatically attract attention (Ochman, Flykt, & Esteves, 2001; Rothermund, Wentura, & Bak, 2001), and that this attraction is disturbed when the amygdala is damaged (Anderson & Phelps, 2001).

To examine the attention-mediation hypothesis of EEM, that is, the enhanced attention allocation to emotional stimuli, Talmi et al. (2008) directly manipulated the way in which attention was allocated during encoding. Participants viewed emotional and neutral stimuli under attention conditions that were either “high”, requiring greater allocation of attentional resources (decision which side of the stimulus has more information), or “low”, requiring lower allocation of attentional resources (detection of the stimulus). In both conditions, encoding was incidental, insofar as participants were not informed about subsequent retrieval. Significantly better recognition of emotional stimuli than neutral stimuli was observed in the “low” attention condition. A similar trend was observed in “high” attention condition. Greater EEM in a condition of low attention during encoding was also observed in studies reported by Kensinger and Corkin (2004) and by Talmi et al. (2007).Encoding of emotional information seems to depend less on voluntary processing than encoding of neutral information, and therefore it requires less resources and attention. Recently, Talmi and McGarry (2012) suggested that immediate EEM could be explained by three cognitive factors: attention (emotional stimuli capture more attention than neutral stimuli), organization (semantic inter-relatedness of a stimulus set), and distinctiveness (the context in which they are embedded, composition of the experimental stimulus set). According to these authors, emotional stimuli are better retrieved because they are better organized, are more distinctive, and attract more attention. When all these factors were controlled in their study, the EEM disappeared.

The question of how processing requirements may modulate effects of emotion on memory was also addressed by manipulating instructions about subsequent retrieval (D’Argembeau & Van der Linden, 2004). The study by D’Argembeau and Van der Linden investigated how the emotional meaning of stimuli influences the learning of contextual information, particularly perceptual information such as color. In addition, how intention to learn modulates this influence was also examined in this study. To examine this effect, they manipulated the intention to learn the information by either instructing subjects to learn it (intentional encoding) or not (incidental encoding). They observed that the intention to learn influenced subjects’ memory of contextual information as regards emotional but not neutral stimuli. Better recall of color in which words were typed was observed for emotional words than for neutral words only after incidental learning. According to D’Argembeau and Van der Linden (2004), the influence of emotional stimulus meaning on contextual memory involves an automatic modulating effect, with, for example, attention automatically being attracted by the emotional stimuli, rather than a voluntary use of attention resources. Interestingly, recognition of emotional stimuli was better than recognition of neutral stimuli, irrespective of the encoding condition. The study by D’Argembeau and Van der Linden (2004) suggests that the effort made to encode the information modulates how the emotional meaning of stimuli affects the learning of contextual information. It also suggests that how emotion affects recognition of the stimuli themselves does not depend on the intention to encode them. Kensinger et al. (2007) reported similar results. They also manipulated the voluntary versus automatic engagement of the allocation of attentional resources either by giving participants the instructions about the subsequent memory test in a reality-monitoring paradigm or by withholding such instructions. They observed no variation in emotional memory enhancement depending on the encoding type. However, it has been suggested that emotion may attract attention to visual properties of the stimulus and in this way may enhance our memory of these stimuli (Adolphs, Denburg, & Tranel, 2001; Kensinger & Schacter, 2007). For example, in their fMRI study, Kensinger and Schacter (2007) showed that increased amygdala activity corresponded to the successful recognition of negative but not of neutral stimuli (in particular, to the retrieval of visual details of these stimuli) and was linked to correct but not incorrect recognition. In the studies by D’Argembeau and Van der Linden (2004) and by Kensinger at al. (2007), the stimuli were words which did not have the same amount and kind of visual properties as pictures. Their visual complexity is poorer as compared to pictures. Accordingly, if the automatic attraction of attention to emotional stimuli has to do with the visual properties of the stimuli, it is possible that, unlike with words, voluntary (intentional) versus automatic (incidental) engagement of attention differently modulates how emotion affects picture recognition because pictures contain many visual details. As far as we know, there is little evidence about whether immediate EEM for pictures is modulated by the nature of engagement of attention (intentional vs. incidental).

Thus, in the present study we aimed to examine whether the presence of the EEM after a short delay between encoding and retrieval depends on the intention to encode the pictorial stimuli. To manipulate the intention to encode we used two conditions: intentional and incidental, and participants were divided into two experimental groups according to these two encoding conditions. Before starting a categorization task (living/non living), participants were explicitly asked to memorize items and were informed about subsequent retrieval (“intentional encoding”) or not (“incidental encoding”). If EEM is due to automatic processing of the emotional content of the stimulus rather than to its voluntary encoding we would expect to observe better re-cognition of emotional than of neutral stimuli after incidental encoding. In the case of intentional encoding, this effect may diminish or disappear because when participants voluntarily focus their attention on the stimuli and make an effort to memorize them they allocate the same amount of attentional resources to both emotional and neutral stimuli.

Our second concern in this study was whether EEM depends not only on the encoding condition but also on retrieval condition. Most evidence for emotional enhancement of memory comes from studies with explicit, intentional retrieval (e.g., Doerksen & Shimamura, 2001; Hamann, 2001). Some researchers have also observed EEM in implicit, incidental retrieval (Arntz, De Groot, & Kindt, 2005; LaBar et al., 1995), although its presence in implicit retrieval needs to be confirmed. Ramponi, Handelsman, and Barnard (2010) recently examined EEM in “conceptual” implicit retrieval after a short time delay. Participants taking part in their study performed incidental encoding of emotional and neutral compound associates words what was followed about 7 min later by either implicit (“Report the first word that comes to mind that is associated with the cue”) or explicit retrieval (“Retrieve the associated word from the study phase”). Better performance with emotional than with neutral compounds was observed only in explicit retrieval. The authors interpreted these findings as evidence that reinstatement of the episodic context is necessary in mediating the emotion effect. Ramponi, Barnard, Kherif, and Henson (2011) replicated these results in an imaging study.

To have a better look at how the nature of retrieval influenced the presence of EEM after a short-delay retrieval, we used two conditions of retrieval. In the explicit retrieval condition, participants were asked to distinguish items presented to them during encoding from new items that were not presented during initial encoding. In the implicit retrieval condition, they again had to categorize “old” items (presented to them during encoding) and new items. In this task they were just told that they would continue the categorization task. When instructed about the subsequent retrieval, the participants were expected to process the stimuli deeper than when they were not informed about the retrieval. In addition, during recognition task they were expected to voluntarily retrieve items presented during the initial encoding phase. By contrast, they were not expected to do so during the second categorization task. If EEM in immediate and after short-delay retrieval depends on the explicit reinstatement of the episodic context, we ought to observe it only in the recognition task, as remembering the context of encoding is not relevant for categorizing items into their semantic category. Thus, independently of their emotional valence, the categorization of previously seen (“old”) stimuli should be faster than that of new stimuli, as observed in priming studies (e.g., Dell’Acqua & Grainger, 1999; Thompson-Schill & Gabrieli, 1999). If EEM in immediate memory and after a short-delay retrieval is not dependent on the explicit reinstatement of the episodic memory we should observe EEM after incidental encoding in both tasks, recognition and categorization.

## Methods

### Participants

Eighty participants (68 women and 12 men), all of them students at the University of Lyon 2, took part in this study. Their age ranged from 18 to 33 with a mean age of 21.2 years (*SD* = 3.5). All had normal or corrected-to-normal vision and they all gave their written informed consent to take part in the study. The study was approved by an ethical committee. The participants were randomly assigned to one of the two experimental groups corresponding to different types of encoding phase (intentional, incidental). Forty participants (32 women and eight men) with a mean age of 19.95 years (*SD* = 2.58) were included into intentional encoding group. The other 40 participants (36 women and four men) with a mean age of 20.25 years (*SD* = 3.20) were included into incidental encoding group.

### Stimuli

One hundred and twenty stimuli were selected from a set of 300 stimuli previously pre-tested separately for emotional valence and arousal in a pilot study involving 42 subjects, on a scale ranging from 1 (*very negative for valence evaluation, not at all arousing for arousal evaluation*) to 7 (*very positive, highly arousing*). In the pilot study, the participants saw all the pictures (presented in random order). The stimuli were colour photographs (4.5 × 4.5 cm) of common living and non-living objects (see Appendix A for examples). They were divided into two lists, List 1 and List 2, each containing 60 stimuli. The lists were compiled in such a way that they both contained the same number of stimuli in terms of their emotional valence (20 negative, 20 neutral, 20 positive) and semantic category (30 living, 30 non-living). To ensure that the emotional stimuli did not differ in terms of their visual complexity, we asked 10 participants to evaluate them on a scale ranging from 1 (*very low complexity, visually simple*) to 5 (*very high complexity, visually complex*). A one-way analysis of variance (ANOVA) performed separately for each list with Emotional Valence (negative, neutral, positive) as repeated factor showed no significant difference in visual complexity between the three types of emotional stimuli (for both lists *p* > .05). As slight differences in luminance may attract attention and, therefore, influence the interpretation of results in terms of emotional valence, the luminance of each photo was determined with the help of a Minolta LS-110 photometer based on three successive measurements. The mean luminance was calculated for the negative, neutral, and positive stimuli for each list separately, and one-way ANOVA was performed to check whether they were similar. There was neither a significant difference between the three types of emotional stimuli for List 1 nor for List 2 (both *p*s ≥ .4).

Each list of 60 stimuli was divided into two sets of 30 stimuli that were used alternately for the encoding phase, whereas all stimuli were used for the retrieval phase. Thus, during the encoding phase, half of the participants saw one set of stimuli and the other half saw the other set. Table 1 shows the distribution of the stimuli and their mean value in terms of emotional valence and arousal as established during a pilot evaluation. Pictures were selected according to the following criteria concerning valence and arousal: mean ratings for negative valence had to be less than or equal to 3.0, mean ratings for positive valence had to be larger than or equal to 5.5, and mean ratings for neutral pictures had to be between 3.5 and 4.5. The mean ratings for arousal for the three kinds of emotional stimuli had to be between 2 and 6. To ensure that the mean valence of the three classes of emotional stimuli was significantly different, we performed t-tests. This was also done for arousal. The results of these comparisons are shown in Table 2. For both lists, the mean emotional valence of negative stimuli was significantly different from the mean valence of both neutral and positive stimuli. The mean valence of neutral stimuli was also significantly different from that of positive stimuli. As regards arousal, there was no difference with regard to either list between the mean arousal of negative and positive stimuli. Negative and positive stimuli were thus equally arousing, and they were both more arousing than neutral stimuli.

**Table 1.** Mean Emotional Valence and Arousal of Stimuli Selected for Lists 1 and 2 Used in Experimental Tasks.

	List1^a^						List 2^a^					
Task	Encoding^b^			Retrieval^c^			Encoding^b^			Retrieval^c^		
Emotional valence	Negative^d^	Neutral^d^	Positive^d^	Negative^d^	Neutral^d^	Positive^d^	Negative^d^	Neutral^d^	Positive^d^	Negative^d^	Neutral^d^	Positive^d^
Mean valence	2,4(0,32)	4,1(0,28)	5,8(0,29)	2,4(0,36)	4,1(0,31)	5,8(0,31)	2,3(0,39)	4,0(0,23)	5,9(0,38)	2,3(0,43)	4,1(0,20)	5,8(0,35)
Mean arousal	4,9(0,67)	4,4(0,84)	4,6(0,68)	5,1(0,58)	3,8(1,0)	4,7(0,62)	5,1(0,54)	3,6(0,91)	4,7(0,64)	5,0(0,67)	3,3(1,0)	4,6(0,63)

*Note*. Standard deviations in parentheses. ^a^60 stimuli. ^b^30 stimuli (15 living/15 non living). ^c^30 stimuli from encoding + 30 new stimuli = total 60 stimuli (30 living/30 non living). ^d^10 stimuli.

**Table 2.** Paired *t*-Tests and *p* Values for Comparisons of Emotional Valence and Arousal Between Negative, Neutral, and Positive Stimuli From Lists 1 and 2.

	List 1^a^				List 2^a^			
Task	Encoding^b^		Retrieval^c^		Encoding^b^		Retrieval^c^	
	Valence	Arousal	Valence	Arousal	Valence	Arousal	Valence	Arousal
Negative vs. neutral	*t*(1, 9) = 12,1 *p* < .000001	*t*(1, 9) = 1,9 p = .09	*t*(1, 19) = 16,1 *p* < .0000001	*t*(1, 19) = 4,5 *p* < .0003	*t*(1, 9) = 16,6 *p* < .0000001	*t*(1, 9) = 6,5 *p* < .0002	*t*(1, 19) = 19,4 *p* < .0000001	*t*(1, 19) = 7,5 *p* < .0000001
Positive vs. neutral	*t*(1, 9) = 11,7*p* < .000001	*t*(1, 9) = 1,0p = .33	*t*(1, 19) = 18,1*p* < .0000001	*t*(1, 19) = 4,2*p* < .0005	*t*(1, 9) = 18,5*p* < .0000001	*t*(1, 9) = 5,4*p* < .0005	*t*(1, 19) = 20,8*p* < .0000001	*t*(1, 19) = 5,1*p* < .00007
Negative vs. neutral	*t*(1, 9) = 21,7 *p* < .000001	*t*(1, 9) = 1,2 p = .25	*t*(1, 19) = 36,4 *p* < .0000001	*t*(1, 19) = 1,8 *p* < .09	*t*(1, 9) = 22,1 *p* < .0000001	*t*(1, 9) = 1,7 *p* < .13	*t*(1, 19) = 33,4 *p* < .0000001	*t*(1, 19) = 1,9 *p* < .07

^a^60 stimuli. ^b^30 stimuli. ^c^30 stimuli from encoding + 30 new.

To check whether the mean emotional valence was the same for the two lists, we performed an ANOVA with factors being List (List 1, List 2) and Stimulus (negative, neutral, positive). Overall, the two lists did not differ in terms of their emotional valence, *F*(1, 9) = 1.52, *p* = .28. There was no significant interaction between List and Stimulus, *F*(2, 18) = .46, *p* = .64. We also checked whether the two lists were equivalent in terms of their emotional arousal. We found there was no significant effect of list, *F*(1, 9) = 1.32, *p* = .28, but the interaction between List and Stimulus was significant, *F*(1, 18) = 8.45, *p* < .0004, due to the fact that the neutral stimuli from List 1 were more arousing than the neutral stimuli from List 2 (p .008). However, the mean arousal of the neutral stimuli from Lists 1 and 2 was significantly lower than the mean arousal of the negative and positive stimuli from the corresponding list (see Table 1). The mean arousal of negative and positive stimuli from List 1 was not significantly different from the mean arousal of the stimuli from List 2 (for negative *p* = .9, and for positive *p* = .54).

### Tasks

Two types of tasks were used in this study: a categorization and a recognition task. In the categorization task, participants had to decide as quickly and as accurately as possible whether the stimulus belonged to the living or to the non-living category. The categorization task was always used in the encoding phase and in the implicit retrieval phase. In the encoding phase, participants were presented with 30 stimuli, whereas in the implicit retrieval phase, they were presented with 60 stimuli, 30 from the encoding phase and 30 new stimuli.

We varied instruction as a way of manipulating the nature of encoding (intentional vs. incidental). For intentional encoding, participants were told they would subsequently be asked to retrieve the presented stimuli among other new stimuli. They were asked to pay attention to the stimuli and to try to memorize them. For incidental encoding, they were not informed about the subsequent retrieval.

In the recognition task, participants had to decide as quickly and as accurately as possible whether or not they had seen the stimulus in the previous task. The recognition task was used only in the explicit retrieval phase. As in the implicit retrieval, participants viewed 60 stimuli, 30 from the encoding phase (categorization task) and 30 new stimuli.

**Figure 1.**
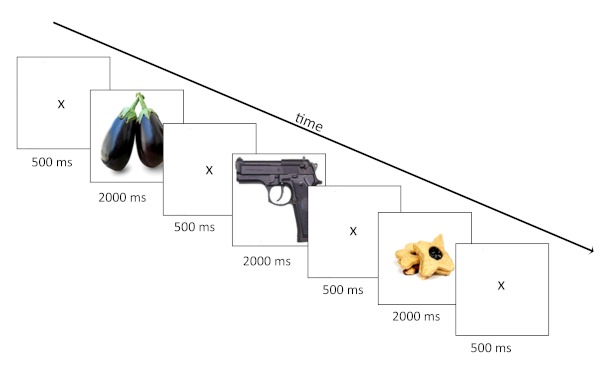
Task method. During encoding and retrieval tasks the stimuli were presented in the same way, as illustrated. During encoding participants decided whether the item belongs to the living or non-living semantic category (categorization task). During explicit retrieval they decided whether the item was seen during encoding task or was not seen (recognition task). During implicit retrieval they performed again categorization task.

In all the tasks the stimuli were presented in the same way, for 2,000 ms in the middle of the computer screen against a white background. Each stimulus appeared immediately after a fixation cross which was displayed on the screen for 500 ms (see Figure 1).

### Procedure

**Figure 2.**
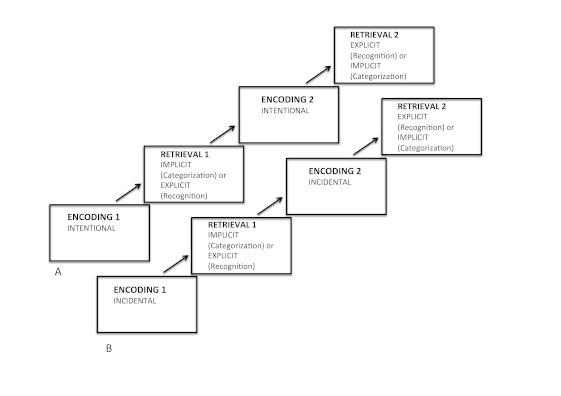
Experimental procedure for two experimental groups. A. Intentional encoding (participants were informed about a following retrieval task). B. Incidental encoding (participant were not informed about a following retrieval task). In each group participants performed two encoding and two retrieval tasks. For half of the participants the first retrieval task was implicit and the second was explicit, it was in a revers way for the other half of the participants.

Each participant performed two encoding phases (always categorization task) and two retrieval phases (categorization task and recognition task; see Figure 2). The only difference between the two experimental groups (intentional/incidental) was the nature of the encoding phase. For the intentional encoding group, both encoding phases were intentional (participants were informed about the subsequent retrieval and asked to make an effort to memorize them). For the incidental encoding group, both encoding phases were incidental (participants were not informed about the subsequent retrieval). The tasks were programmed and run using DMDX software (Forster & Forster, 2003).

Before the experimental session started, each participant signed an informed consent form regarding his/her participation in the study. The participants were seated in a quiet room with a laptop computer in front of them, at a distance of 40 cm. They were informed that the test would take 30 min and that they would be required to perform several tasks for which they would receive instructions in due course.

The participants always started with the encoding phase (the categorization task) lasting approximately 2.5 min. For both groups, the encoding phase was immediately followed by the implicit retrieval phase with a corresponding list (categorization task) lasting approximately 4.5 min. Half of the participants responded by hitting the shift key on the right-hand side of the keyboard for non-living stimuli, and on the left-hand side for living stimuli, the other half responded in the revers way. Their accuracy and reaction times (RTs) were recorded by the computer. At the end of the retrieval phase, participants were asked to take part in a distractive task. They were required to silently read a short text (20 lines passage from the Encyclopaedia describing French geography) and then answer two questions about the text. The task took about 10 min. A second encoding phase ensued (with a second list of stimuli) and was immediately followed by the explicit retrieval phase (recognition task). Half of the participants responded by hitting the shift key on their right-hand side of the keyboard (previously unseen stimuli: new) and the shift key on the left-hand side (previously seen stimuli: old), the other half did it in the reverse way.

At the end of the second retrieval phase, participants were thanked for their participation, and the experimenter gave them explanations about the purpose of the study. The order of the presentation of the Lists 1 and 2 was counterbalanced between subjects.

## Results

### Explicit retrieval (recognition task)

A mean score of correct recognition (hits) and false alarms (FAs) is presented in Table 3 as a function of emotional valence (negative, neutral, and positive) and type of encoding (incidental, intentional). Six of the participants were discarded from the statistical analysis because of ceiling effects (their hits scores were 100% correct).

**Table 3.** Recognition Performance (Mean Number of Correct Recognitions and False Alarms) as Function of Encoding Type and Emotion.

	Hits		FAs	
Encoding condition and stimulus type	*M*	*SE*	*M*	*SE*
Intentional				
Negative	8,62	0,23	1,38	0,18
Neutral	8,46	0,21	1,24	0,16
Positive	8,57	0,18	1,51	0,23
Incidental				
Negative	9,00	0,18	1,54	0,28
Neutral	8,49	0,23	1,14	0,18
Positive	9,10	0,15	0,95	0,17

*Note*. Hits = correct recognitions. FAs = false alarms. The maximum score for hits and FAs in each experimental condition was 10.

The index of sensitivity (*d*’) and response criterion (C) were analysed with a two-way mixed measures ANOVA with group factor being Encoding (intentional, incidental) and one repeated-measures factor being Emotional Valence (negative, neural, and positive).

**Figure 3.**
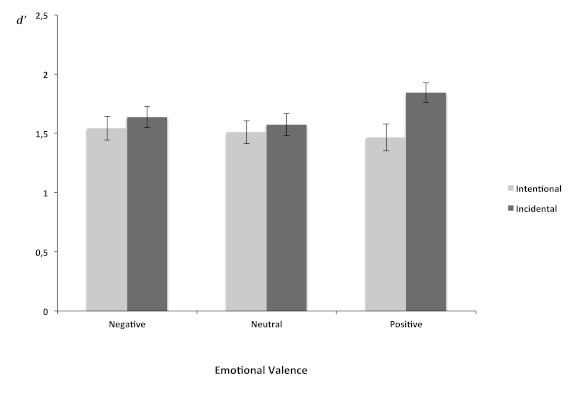
Mean value of *d*’ index (±1*SEM*) in recognition task for negative, neutral and positive stimuli after intentional and incidental encoding.

As far as *d*’ was concerned, the effects of emotional valence, *F*(2, 144) = 1.39, *p* = .25, and Encoding, *F*(1, 72) = 2.57, *p* = .12, were not significant. However, the interaction between Encoding and Emotional Valence was significant, *F*(2, 144) = 3.29, *p* < .04 (see Figure 3). Multiple comparisons showed that the capacity to discriminate between old and new stimuli was better for positive (*d*’ = 1.84) than for neutral (*d*’ = 1.57, *p* < .003) or for negative stimuli (*d*’ = 1.64, *p* < .04) after incidental encoding. There was no significant difference between negative and neutral stimuli (*p* = .5). No significant difference was observed in the capacity to discriminate between positive, neutral, and negative stimuli after intentional encoding: positive (*d*’ = 1.47) versus neutral (*d*’ = 1.51, *p* = .6) and negative stimuli (*d*’ = 1.54, *p* = .4); neutral versus negative stimuli (*p* = .7).

**Figure 4.**
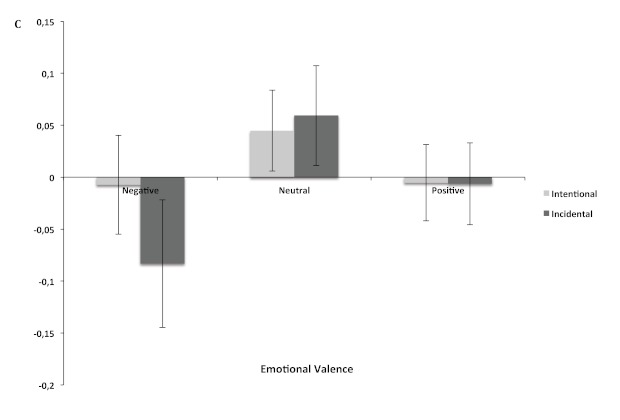
Mean value of *C* index (±1*SEM*) in recognition task for negative, neutral and positive stimuli after intentional and incidental encoding.

As far as the response criterion was concerned, there was no significant effect of encoding, *F*(1, 72) = 0.24, *p* = .64, and the interaction between Encoding and Emotional Valence was not significant, *F*(1, 72) = 0.64, *p* = .53 (see Figure 4). The effect of emotional valence was close to significance level, *F*(1, 72) = 2.62, *p* = .07. Multiple comparisons showed that participants’ response criterion was significantly more liberal for negative (*C* = -.05) than for neutral stimuli (*C* = .05, *p* < .03). There was no significant difference between negative and positive stimuli (*C* = -.005, *p* = .35), and between positive and neutral stimuli (*p* = .19).

### Implicit retrieval (categorization task)

For this task we performed the analyses only on RTs because participants had to categorize items again and not to recognize them.

**Figure 5.**
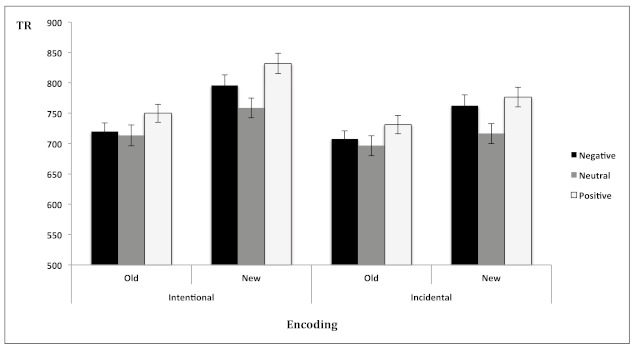
Mean reaction times (±1*SEM*) for old and new stimuli as a function of emotional valence (negative, neutral and positive) and encoding (intentional, incidental).

A two-way mixed ANOVA was carried out on mean correct RTs with the between-subjects factor being Encoding (intentional vs. incidental) and the repeated-measures factors being Emotional Valence (negative, neutral, and positive) and Stimulus (old, new). This ANOVA showed a significant effect of stimulus, *F*(1, 78) = 104.8, *p* < .0001. The participants responded significantly faster to old (*M* = 717 ms, *SE* = 22) than to new items (*M* = 774 ms, *SE* = 24). The effect of emotional valence was also significant, *F*(2, 156) = 29.21, *p* < .0001. Participants responded significantly faster to neutral (*M* = 721 ms, *SE* = 16) than to negative, *F*(1, 78) = 12.53, *p* < .0007; *M* = 744 ms, *SE* = 15, and positive stimuli, *F*(1, 78) = 56.09, *p* < .0001; *M* = 772 ms, *SE* = 15. They also responded faster to negative than to positive stimuli, *F*(1, 78) = 16.3, *p* < .0002. The effect of encoding was not significant, *F*(1, 78) = 1.15, *p* = .29). In the recognition task after intentional encoding (*M* = 761 ms, *SE* = 26), participants were as fast as after incidental encoding (*M* = 729 ms, *SE* = 19). An interaction between Stimulus and Emotional Valence was also significant, *F*(2, 156) = 5.25, *p* < .007. For old stimuli, planned comparisons showed that participants responded significantly slower to positive than to negative (*p* < .0005) and neutral stimuli (*p* < .0009). On the contrary, there was no significant difference between old negative and old neutral stimuli (*p* = .76; see Figure 5). For new stimuli participants responded significantly slower to positive (*p* < .0001) and negative stimuli (*p* < .0003) than to neutral stimuli. They also responded significantly slower to positive (*p* < .02) than to negative stimuli. The planned comparisons between negative old and negative new (*p* < .0001), neutral old and neutral new (*p* < .0003), and positive old and positive new stimuli (*p* < .0001) were all significant, with new stimuli being categorized slower than old stimuli. There was no other significant interaction.

In order to better understand the effect of emotion on memory, we also analysed priming effects, that is, the difference in RTs between old and new items with a two-factor mixed ANOVA, with the group factor being Encoding (intentional, incidental) and with the repeated-measures factor being Emotion (negative, neutral, positive). The effect of group was significant, *F*(1, 78) = 4.64, *p* < .04. Priming effects after intentional encoding were larger (*M* = 68 ms, *SE* = 7.7) than after incidental encoding (*M* = 44 ms, *SE* = 7.7). The effect of emotion was also significant, *F*(2, 156) = 5.26, *p* < .007, since, compared to neutral stimuli (*M* = 32 ms, *SE* = 8.6), negative stimuli (*M* = 72 ms, *SE* = 9.2, *p* < .06) and positive stimuli (*M* = 63 ms, *SE* = 9.6, *p* < 0.01) yielded larger effects. The interaction between Group and Emotion was not significant, *F*(2, 156) = 0.6, *p* = .56.

Because Lists 1 and 2 significantly differed in mean arousal, we checked whether mean RTs and mean number of correct responses per item correlated with emotional arousal of the items. We did not find any significant correlation, neither in the recognition task, RT: *r*(28) = -.06, *p* = .73; correct responses: *r*(28) = .33, *p* = .08; nor in the categorisation task, RT: *r*(28) = .31, *p* = .11; correct responses: *r*(28) = -.21, *p* = .29.

Because RTs for positive stimuli were slower in the categorisation task and the sensitivity for these stimuli was higher in the recognition task, we examined whether there is a correlation between them. There was no significant correlation, *r*(37) = .18, *p* = .75, suggesting that there is no link between these two effects.

To summarize, as indexed by *d*’, after incidental encoding, recognition of positive stimuli was easier than that of neutral and negative stimuli. The participants’ response criterion was more liberal for negative than for neutral stimuli independently of the encoding condition.

In the implicit retrieval task, participants responded faster for old than for new stimuli independently of their emotional valence. They responded slower for positive than for neutral and negative stimuli after both intentional and incidental encoding. However, the priming effect was larger for both positive and negative stimuli than for neutral ones.

## Discussion

In the present study we investigated the effects of emotion value of pictorial stimuli on implicit and explicit retrieval after a short time delay. Especially, we were interested to see whether these effects depend on the intention to encode information.

In line with the proposal that the influence of emotion on memory is based on the automatic, involuntary attraction of attention during encoding (D’Argembeau & Van der Linden, 2004, 2005), we expected to observe EEM only after incidental encoding where allocation of the attentional resources is rather automatic. On the contrary, during intentional encoding where the allocation of attention is rather voluntary, the same amount of attentional resources would be allowed to emotional and neutral stimuli. In addition, based on the assumption that reinstatement of the episodic context is necessary to mediate the EEM (Ramponi et al., 2010), better performance was expected with emotional stimuli than with neutral stimuli, especially in a recognition task where retrieval was explicit. If the mediating effects of emotion on immediate memory do not depend on the reinstatement of the episodic context, better performance with emotional stimuli should also be observed in the case of implicit retrieval after incidental encoding.

As indicated by *d*’ index, we observed that participants discriminated positive old from new stimuli after incidental encoding, but not after intentional encoding. This was not the case for negative stimuli. In fact, *d*’ was higher for positive than for neutral stimuli, but no significant difference was observed between negative and neutral stimuli. D’Argembeau and Van der Linden (2004) suggested that emotional stimuli attract attention to their perceptual details when the information is learned incidentally, to explain why recognition after incidental encoding of contextual information was better for emotional stimuli than for neutral stimuli. They proposed that insofar as emotional stimuli do not induce strong emotional arousal, they automatically attract attention to the contextual and perceptual details and consequently enhance memory for this information. The stimuli used in the present study were pictures of isolated objects, and were unlikely to induce strong arousal. This was confirmed by performing a pre-test on our stimuli. Thus, the better discrimination for positive stimuli in the current study may be due to the fact that, during encoding, attention was drawn to the details of these stimuli more than to those of neutral and negative stimuli. D’Argembeau and Van der Linden (2004) observed no influence of the encoding condition on the recognition of the emotional stimuli themselves. However, the stimuli in their study were words. Unlike words, the present study’s emotional pictures contain many visual details to which attention may be automatically attracted. This in turn may have enhanced memory as a result of increased processing of these details. The data we observed for the new stimuli in the categorisation task lend support to this idea. We found that new emotional stimuli were categorized more slowly than neutral stimuli. The slower RTs for emotional stimuli may be due to the fact that participants took more time to explore their perceptual details, for example, because they automatically attracted their attention.

The better discrimination for positive stimuli, indicated by higher d’, but not negative stimuli, that we observed, fits well with Talmi et al.’s (2007) proposition that positive stimuli garner extra attention during encoding, and that this contributes to the effects of positive emotion on memory. The authors suggested that EEM for positive stimuli is due to the allocation of attention to these stimuli. It is also possible that moderately-arousing emotional stimuli, as the ones used here, benefit from the additional recruitment of controlled processes during encoding (Kensinger & Corkin, 2004). Participants may spontaneously elaborate or rehearse positive emotional stimuli more than neutral stimuli. This may provide an account for the data observed in the present study. When the participants are explicitly asked to memorise the stimuli, this spontaneous elaboration and rehearsal may decrease, and a similar amount of resources may be involved in the processing of emotional and neutral stimuli. Talmi et al. (2007) suggested that emotional stimuli are more semantically related to each other than neutral ones, and this may account for part of the EEM. Yet, such an explanation cannot account for the disappearance of the effect after intentional encoding. Talmi et al. (2007) proposed that EEM for negative stimuli is rather due to their arousal then to the allocation of attention or semantic relatedness. It is possible that we did not observe EEM for negative stimuli because these were not arousing enough.

The task that we used during encoding may suggest another explanation for the absence of EEM for negative stimuli. Our categorization task was similar to Talmi et al.’s (2008) “high-attention” encoding condition in which the effects of emotion were weak. Nevertheless, participants used a more liberal response criterion for negative stimuli than for neutral stimuli, but not for positive ones. They were more willing to say that they saw a negative item during encoding, suggesting that negative stimuli influence retrieval strategies. These results partly agree with those of Dougal and Rotello (2007) who suggested that a more liberal criterion is used for emotionally negative stimuli.

More recently, Talmi and McGarry (2012) suggested that concerning moderately arousing negative stimuli EEM may be completely explained by three cognitive factors, primary distinctiveness (composition of the experimental stimulus set), organization (semantic cohesiveness or inter-relatedness), and attention (emotional stimuli capture more attention than neutral stimuli). According to these authors, emotional stimuli are better retrieved because they are better organized, are more distinctive, and attract more attention. When all these factors were experimentally controlled for, the EEM in Talmi and McGarry’s study disappeared. In the present study, primary distinctiveness of the stimuli was not controlled for, emotional and neutral stimuli were intermixed, and the same number of stimuli of each emotional category was presented, so the retrieval should have been better for negative stimuli. However, negative and neutral stimuli were semantically related because they belonged to the same semantic categories (living and non-living), so this may be one of the reasons why we did not observe the EEM for negative stimuli.

In the categorisation task we observed effects of priming independent of the emotional valence of stimuli. Participants categorised previously seen items more quickly than new items suggesting they had been encoded. This is in line with previous research (Dell’Acqua & Grainger, 1999; Thompson-Schill & Gabrieli, 1999). In our study, this priming effect was greater for negative and positive stimuli than for neutral stimuli.

We unexpectedly observed some effects of emotion on categorisation time of both old and new items, and independently of encoding type. Participants were slower to categorize both old and new positive stimuli than negative and neutral stimuli. They also categorized new negative stimuli more slowly than neutral ones. However, there was no correlation between *d*’ and RT in the categorisation task for positive stimuli, suggesting that there is no link between the EEM effects in the recognition task and in the categorisation task.

The results observed in the implicit memory test run somewhat counter to the suggestion made by Ramponi et al. (2010) that intentional retrieval is necessary for the effect of emotion on memory. In the present study, participants were not told about the repetition of the stimuli in the second categorization task and were not expected to retrieve them explicitly. However, we did observe an effect of emotion for old and new stimuli. Because we did not check whether the participants were aware that some stimuli were repeated, the possibility that our implicit task was in some way contaminated by that awareness cannot be ruled out completely. However, none of the participants spontaneously reported being aware of the repetition. Because the performance with negative old stimuli did not differ from that with neutral old stimuli, it is not clear why such awareness would only have influenced performance with positive old stimuli. Another explanation for the slower performance with positive old stimuli may be that, as suggested by some authors, negative stimuli attract more attention than positive stimuli and are processed more quickly because they are more important for survival (e.g., Hansen & Hansen, 1994). Accordingly, the suggestion is that there is a stable attentional bias in favour of negative stimuli. However, once again, this explanation does not hold true, since we would expect significantly faster performance for negative old stimuli than for neutral old stimuli, and no such difference was found. Nor does it explain why positive stimuli were categorized more slowly than neutral stimuli. There is another possible explanation for these results. It may be that, in general, positive stimuli were more difficult to categorize than negative and neutral stimuli. However, participants categorised negative, neutral, and positive stimuli equally well, with their performance reaching ceiling levels. Thus, it seems that categorisation was not particularly more difficult for positive stimuli than for negative and neutral pictures.

To summarize, our study suggests that an intention to encode or to not encode information influences the effect of emotion on immediate memory. It also suggests that positive and negative valence of stimuli may have different effects on immediate recognition memory, the first affecting sensitivity and the second influencing a response criterion. In addition, this study provides evidence that EEM for positive stimuli does not depend on the intentionality of retrieval.
